# The genome of an enigmatic sea urchin parasite *Echinomermella matsi* Jones & Hagen, 1987 resolves its place among other invertebrate parasitic nematodes

**DOI:** 10.1093/g3journal/jkag084

**Published:** 2026-04-06

**Authors:** Joseph Kirangwa, Erna King, Joanna Collins, Adam Bates, Mark Blaxter, Oleksandr Holovachov

**Affiliations:** Department of Biochemistry, University of Oxford, Dorothy Crowfoot Hodgkin Building, South Parks Rd, Oxford OX1 3QU, United Kingdom; Tree of Life, Wellcome Sanger Institute, Wellcome Genome Campus, Cambridge CB10 1SA, United Kingdom; Tree of Life, Wellcome Sanger Institute, Wellcome Genome Campus, Cambridge CB10 1SA, United Kingdom; Tree of Life, Wellcome Sanger Institute, Wellcome Genome Campus, Cambridge CB10 1SA, United Kingdom; Tree of Life, Wellcome Sanger Institute, Wellcome Genome Campus, Cambridge CB10 1SA, United Kingdom; Department of Zoology, Swedish Museum of Natural History, Stockholm 104 05, Sweden

**Keywords:** Benthimermithidae, Camacolaimidae, evolution, marine, parasitism, parasitoidism, Spirurina

## Abstract

We present a genome of *Echinomermella matsi* (Nematoda: Plectida: Benthimermithidae), a body cavity parasite of the green sea urchin *Strongylocentrotus* spp. commonly found along the coast of Central and Northern Norway. Three assemblies were generated, 1 from multiple individuals using Oxford Nanopore long-read data and 2 from 2 individuals using PacBio long-read data. The genome of *Echinomermella matsi* is 65 Mb long consisting of 7 chromosomes, with nematode Benchmarking Using Single Copy Orthologue (BUSCO odb12) completeness reaching 96%. The *E. matsi* chromosome complement corresponds to the proposed Rhabditida ancestral linkage groups. Phylogenetic analyses using newly generated 18S rRNA genes and a multigene dataset consisting of BUSCO protein-coding genes, supported by morphological observations of juveniles, firmly place *Echinomermella* within the nematode order Plectida, alongside nematode parasitoids of marine invertebrates, *Trophomera* or *Neocamacolaimus*. As a result, the generally free-living order Plectida includes at least 3 independently evolved lineages of nematodes symbiotic with various groups of aquatic and terrestrial invertebrates and with unicellular organisms. This, and the fact that Plectida is the closest sister lineage to Rhabditida as a whole, and 1 node away from the exclusively animal parasitic Spirurina, makes this lineage a valuable model for study of evolution of animal parasitism in the aquatic environment.

## Graphical Abstract

**Graphical Abstract:**
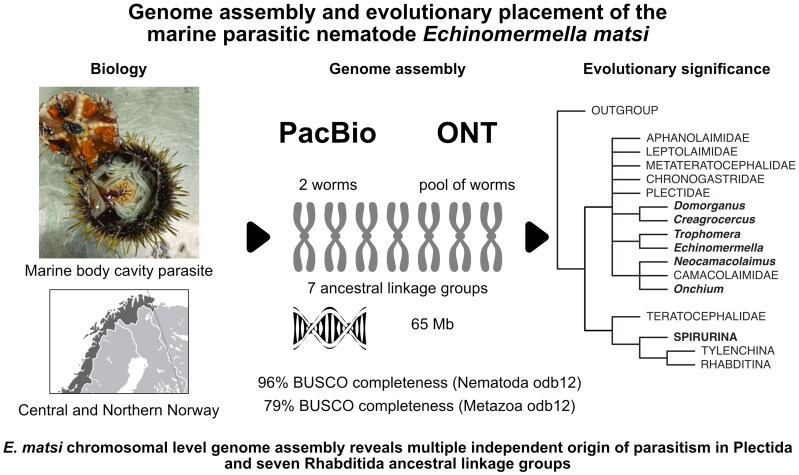

## Introduction

*Echinomermella* Chitwood, 1933 are peculiar and poorly known marine nematodes. They are endoparasites of sea urchins, with only 2 described species. *Echinomermella grayi* (Gemmil 1901) Chitwood, 1933, a parasite of the European edible or common sea urchin *Echinus esculentus*, was first described under the name of *Echinonema grayi* or *Ichthyonema grayi* from the Firth of Clyde in Scotland (Gemmill 1901; Gemmill and von Linstow 1902). The same species was subsequently recorded from the same host from several localities along the coast of Brittany (Roscoff) and the coasts of Britain (Plymouth, Scarborough, Isle of Mull and Slate Islands) and Shetland (Shipley 1901; Barel and Kramers 1977; Comely and Ansel 1988), but its biology and interaction with the host remain poorly known. The second known species of *Echinomermella*, *Echinomermella matsi* Jones & Hagen, 1987 from the body cavity of the green sea urchin *Strongylocentrotus droebachiensis* is known much better. Originally discovered in Vestfjorden (Jones and Hagen 1987), the species is now known to be common along the coast of Central and Northern Norway (Sivertsen 1996), infecting over half of the population of its host in some localities (Stien et al. 1998). Naturally, such impact on a commercial species and its potential economic importance for mariculture (Hagen and Siikavuopio 2010) has not been ignored. The morphology, distribution, ecology, and host–parasite interaction of *E. matsi* have been studied in greater detail (Hagen 1992, 1996; Poinar et al. 2011; Stien 1999; Stien and Halvorsen 1998; Stien et al. 1996). However, the evolutionary relationships of *Echinomermella* with other animal parasitic nematodes remain unclear.

**Fig. 1.**
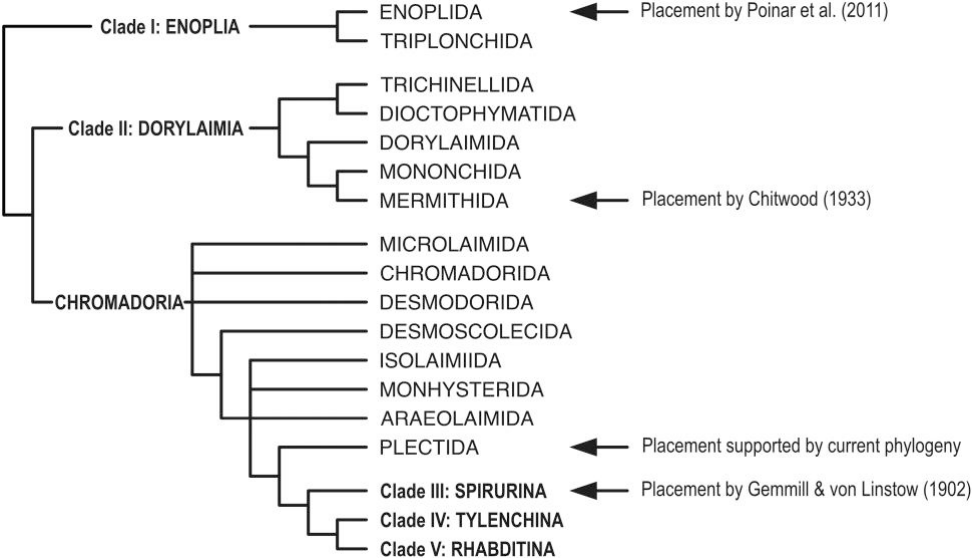
Alternative placements of the genus *Echinomermella* in the simplified consensus nematode phylogeny.

When discovered and described, these nematodes were first placed in the genus *Ichthyonema* (Gemmill and von Linstow 1902), and later in *Philometra* (Yorke and Maplestone 1926), other species of which are parasites of freshwater and marine fish and are currently classified in the family Philometridae (Fig. 1; Spirurina; clade III of Blaxter et al. 1998). Reinterpreting the data from the original description, Chitwood (1933) proposed a new genus name *Echinomermella* and provisionally placed it in Mermithoidea (Fig. 1; equivalent to the order Mermithida, clade I of Blaxter et al. 1998), the notion that was followed by Jones and Hagen (1987). *Echinomermella* has been associated with the parasitic “marimermithids,” a set of marine parasites now known to be polyphyletic (Westerman et al. 2021; Tchesunov et al. 2023; Zograf et al. 2024). Based on phylogenetic analysis of a single partial 18S rRNA sequence of *E. matsi* derived from a formaldehyde-preserved specimen, Poinar et al. (2011) proposed a close relationship between *Echinomermella* and marine free-living predatory *Enoplus* nematodes from the family Enoplidae (Fig. 1; order Enoplida; clade II of Blaxter et al. 1998). Other “marimermithid” taxa have now been placed using 18S ribosomal RNA phylogenetics within 3 distinct families of enoplian nematodes (Enoplia; clade I), within Mermithida (Dorylaimia, clade II) and within Spirurina (Rhabditida; clade III) (Tchesunov et al. 2023). These differences led Tchesunov et al. (2023) to propose that *Echinomermella* could be “a natural model system to study genomic predispositions to parasitism.” We leverage newly sequenced genomes of *E. matsi* to redefine its phylogenetic affinities and deepen our understanding of the origin of nematode parasites of marine invertebrates.

## Material and methods

### Sampling, preservation, and light microscopy

Nematodes (females, males, and juveniles) were collected during the HHUMTL22 cruise onboard R/V Helmer Hanssen (Wernström et al. 2022). Sea urchins of unidentified species of the genus *Strongylocentrotus* were collected by divers in the Langsundet straight near Hansnes, Troms county, Norway (N 69° 57′ 40″; E 19° 37′ 22″) and stored in plastic containers with running seawater until dissection. Sampling did not require any state-issued permits or owner permissions. Nematodes were preserved in RNAlater, in 95% ethanol, and directly frozen at −80 °C without any preservative or storage liquid. Selected specimens from ethanol were processed to absolute glycerin and mounted on permanent glass slides using paraffin as a support for the cover slip (van Bezooijen 2006). Morphological studies were carried out using a Nikon Eclipse 80i microscope equipped with differential interference contrast illumination and a Sony a6400 digital camera.

### Genome sequencing and assembly

#### PacBio and Hi-C data generation and assembly

In the Tree of Life core laboratory, tissue from the midbody of 1 adult female (Tree of Life identifier neEchMats5) and 1 whole small individual, putatively identified as a male (Tree of Life identifier neEchMats11), was homogenized individually using a PowerMasher II tissue disruptor (Denton et al. 2023). High molecular weight DNA was extracted using the Manual MagAttract protocol (Strickland et al. 2023) and purified using AMPure PB SPRI (0.45×) to eliminate shorter fragments and concentrate the DNA. The DNA concentration was assessed using a Nanodrop spectrophotometer and Qubit (dsDNA High Sensitivity Assay kit), and the fragment size distribution was evaluated using the FemtoPulse system. Ultralow input PacBio sequencing was deemed necessary for the yields obtained, and DNA was therefore fragmented using the Covaris g-TUBE method (Oatley et al. 2023) and purified with AMPure PB beads (0.6×). The concentration and fragment size distribution of the sheared and purified DNA were assessed as described previously, and the samples were submitted to WSI Scientific Operations for sequencing on a Pacific Biosciences Revio instrument.

Hi-C libraries were prepared using the Arima-HiC v2 kit. In brief, frozen tissue (stored at −80 °C) was fixed, and DNA crosslinked using TC buffer with 22% formaldehyde. After crosslinking, the tissue was homogenized using the Diagnocine Power Masher-II and BioMasher-II tubes and pestles. Following the kit manufacturer's instructions, crosslinked DNA was digested using a restriction enzyme master mix. The 5′-overhangs were then filled in and labeled with biotinylated nucleotides and proximally ligated. An overnight incubation was carried out for enzymes to digest remaining proteins and for crosslinks to reverse. A cleanup was performed with SPRIselect beads prior to library preparation. For Hi-C library preparation, DNA was fragmented to a size of 400 to 600 bp using a Covaris E220 sonicator. The DNA was then enriched, barcoded, and amplified using the NEBNext Ultra II DNA Library Prep Kit following manufacturers' instructions. The Hi-C sequencing was performed using paired-end sequencing with a read length of 150 bp on an Illumina NovaSeq 6000 instrument.

The *E. matsi* neEchMats5.1 (GCA_964256745.1) and neEchMats11.1 (GCA_964248945.1) genomes were assembled following the Tree of Life Assembly process. Initial PacBio HiFi assemblies were generated with *Hifiasm* (Cheng et al. 2024; v0.19.8-r603, –primary mode). Hi-C reads were aligned against the primary *Hifiasm* assembly using bwa mem (Li 2013; v0.7.17-r1188), PCR duplicates were filtered using picard (https://broadinstitute.github.io/picard/; v2.18.29-0), and scaffolding was conducted with *YaHS* (Zhou et al. 2023; v1.1a). The primary assemblies were analyzed and manually improved using *TreeVal* (https://doi.org/10.5281/zenodo.10047653), and the chromosome-scale scaffolds confirmed by the Hi-C data were named in order of size. The mitochondrial genome was assembled using *OATK* (Zhou et al. 2025).

#### Nanopore data generation and assembly

DNA from several females was extracted using the Zymo Quick-DNA HMW MagBead Kit. A long-read sequencing library was prepared using a ligation sequencing kit SQK-LSK114 (Oxford Nanopore Technologies, Oxford, UK) following the manufacturer's instructions. The prepared library was sequenced with R14 chemistry using MinION Mk1B and FLO-MIN114 flow cell (Oxford Nanopore Technologies, UK), and the reads were basecalled with high accuracy using *Dorado* server postsequencing. *fastp* (Chen et al. 2018), *FastQC* (Andrews 2024), *Meryl* (Rhie et al. 2020), and *GenomeScope* (Ranallo-Benavidez et al. 2020) were used to preprocess raw reads and assess the quality of filtered reads via the *Galaxy|Australia—Genome Lab* interface (The Galaxy Community 2024).

The assembly, called neEchMatsONT (GCA_977018575), was generated using *Flye* (Galaxy version 2.9.5) assembler (Kolmogorov et al. 2019) in Nanopore-HQ mode with 3 polishing iterations. *BlobToolKit* (Challis et al. 2020) was used to identify contigs belonging to putative contaminants and cobionts and to evaluate the final assembly. Hi-C-based scaffolding was conducted with the chromosome conformation capture data generated for the neEchMats5.1 specimen and using the same approaches as PacBio data (see above). Importantly, the Hi-C scaffolding and curation identified illegitimate chimeric joins in the ONT primary assembly that had fused fragments of distinct chromosomes.

### Phylogenetic analysis using 18S rRNA gene

Ribosomal genes were extracted from all 3 genome assemblies of *E. matsi* using *Barrnap* (Seemann 2013) as implemented in *Galaxy|Australia* (The Galaxy Community 2024), aligned and visually compared using *AliView* (Larsson 2014). 18S rRNA from the 3 assemblies were identical. 18S rRNA sequences were compared with other nematode sequences using NCBI (Altschul et al. 1990) and showed no significant similarity to the existing partial 18S rRNA sequence (accession HQ668023) generated by Poinar et al. (2011). To resolve the phylogenetic position of *Echinomermella*, previously published alignments (Ahmed and Holovachov 2021) were used as templates for alignment and annotation of selected nematode sequences spanning the entire phylum, including HQ668023 and the 18S rRNA locus from the 3 *E. matsi* assemblies. Secondary structure annotation was manually added to all nonannotated sequences using *4SALE* (Seibel et al. 2006), and all sequences were manually aligned to maximize apparent positional homology of nucleotides. The phylogenetic tree was inferred with *IQTree* (Minh et al. 2020) using the unpartitioned alignment and built in *ModelFinder* (Kalyaanamoorthy et al. 2017) with the following command: *iqtree2 -s./input.fas -st DNA -m MFP -b 1000 -T AUTO*.

### Phylogenetic analysis using multigene dataset

Protein-coding genes were obtained from the genome-predicted gene sets or translated transcriptomes of 43 species (including all 3 *E. matsi* genomes; Table 1). The protein-coding ortholog genes (translated into amino acids) were extracted from each individual genome or transcriptome assembly using *BUSCO* v5.5.0 (Simão et al. 2015) and the Nematoda orthologs from OrthoDB v10 (nematoda_odb10). A custom script was used to merge identical orthologs (likely to be due to unpurged haplotypic duplication) from the genome datasets into individual orthogroup files (2,365 in total), which were subsequently aligned with *MAFFT* (Katoh et al. 2005) using the *L-INS-i* strategy. Alignments were trimmed with *ClipKIT* (Steenwyk et al. 2020) using default *smart-gap* settings to remove ambiguously aligned regions. To screen candidate core ortholog groups for paralogs and contaminants, following the strategy employed in Smythe et al. (2019) and Ahmed et al. (2022), a maximum-likelihood tree was inferred for each alignment using *IQ-TREE* (2.3.1-macOS-arm version, Minh et al. 2020) with the substitution model automatically selected by *ModelFinder* (Kalyaanamoorthy et al. 2017) for each alignment, followed by 1,000 bootstrap pseudoreplicates. Next, *PhyloPyPruner* (Thalen and Kocot 2024) was used to screen each candidate ortholog for evidence of paralogy with the following settings: *–trim-lb 5 –min-support 0.75 –prune MI –mask pdist –min-taxa 25*, removing putative paralogs and contaminants and retaining only alignments with 25 or more terminal taxa. The remaining 1,308 alignment files were used to infer maximum-likelihood gene trees with the same settings as above, using *IQ-TREE* (2.3.1-macOS-arm version, Minh et al. 2020) and substitution model automatically selected by *ModelFinder* (Kalyaanamoorthy et al. 2017), followed by 1,000 bootstrap pseudoreplicates. Coalescent-based phylogeny was inferred by combining all 1,308 gene trees using *weighted ASTRAL* (*wASTRAL*) option of *ASTER* (Zhang and Mirarab 2022), considering clades with bootstrap support 70% and higher.

**Table 1.** Species included in the phylogenomic analysis.

Name	BioProject/SRA accessions	Data type	BUSCO Nematoda odb10 completeness	Source
Enoplida				
* Enoplus brevis*	PRJEB7588	Transcriptome	C: 61.2% [S: 21.4%, D: 39.8%], F: 2.1%, M: 36.7%	Koutsovoulos (2015)
* Thoracostomopsis barbata*	SRR16481605	Transcriptome	C: 39.1% [S: 19.6%, D: 19.5%], F: 4.6%, M: 56.3%	Tchesunov et al. (2023)
* Enoplolaimus lenunculus*	PRJNA953805	Genome	C: 39.3% [S: 36.1%, D: 3.2%], F: 4.5%, M: 56.2%	Lee et al. (2023)
* Pseudocella trichodes*	SRR16481604	Transcriptome	C: 56.7% [S: 15.9%, D: 40.8%], F: 3.4%, M: 39.9%	Tchesunov et al. (2023)
* Thoracostoma* sp.	PRJNA772260	Transcriptome	C: 54.3% [S: 14.4%, D: 39.9%], F: 3.1%, M: 42.6%	Tchesunov et al. (2023)
* Pontonema vulgare*	PRJNA504396	Transcriptome	C: 62.3% [S: 46.4%, D: 15.9%], F: 1.7%, M: 36.0%	Smythe et al. (2019)
* Trissonchulus latispiculum*	PRJNA953805	Genome	C: 35.6% [S: 26.8%, D: 8.8%], F: 6.0%, M: 58.4%	Lee et al. (2023)
Triplonchida				
* Tobrilus* sp.	PRJNA506158	Transcriptome	C: 50.1% [S: 31.3%, D: 18.8%], F: 3.2%, M: 46.7%	Smythe et al. (2019)
* Tripyla cf glomerans*	SRR16481603	Transcriptome	C: 48.8% [S: 14.5%, D: 34.3%], F: 4.4%, M: 46.8%	Tchesunov et al. (2023)
Mononchida				
* Prionchulus punctatus*	PRJEB7585	Transcriptome	C: 52.0% [S: 34.0%, D: 18.0%], F: 6.2%, M: 41.8%	Koutsovoulos (2015)
Mermithida				
* Romanomermis culicivorax*	PRJEB66727	Genome	C: 39.4% [S: 37.3%, D: 2.1%], F: 4.1%, M: 56.5%	Guiglielmoni et al. (2024)
* Mermis nigrescens*	PRJNA802644	Genome	C: 40.1% [S: 35.5%, D: 4.6%], F: 4.4%, M: 55.5%	Bhattarai et al. (2024)
Dorylaimida				
* Longidorus elongatus*	PRJEB8328	Transcriptome	C: 61.3% [S: 23.8%, D: 37.5%], F: 3.7%, M: 35.0%	Danchin et al. (2017)
* Mesodorylaimus* sp.	PRJNA953805	Genome	C: 52.4% [S: 45.9%, D: 6.5%], F: 5.1%, M: 42.5%	Lee et al. (2023)
Dioctophymatida				
* Soboliphyme baturini*	PRJEB516	Genome	C: 35.5% [S: 34.8%, D: 0.7%], F: 6.2%, M: 58.3%	International Helminth Genomes Consortium (2019)
Trichinellida				
* Trichuris muris*	PRJEB126	Genome	C: 56.2% [S: 55.1%, D: 1.1%], F: 2.5%, M: 41.3%	Foth et al. (2014)
* Trichinella spiralis*	PRJNA257433	Genome	C: 68.5% [S: 38.9%, D: 29.6%], F: 0.7%, M: 30.8%	Korhonen et al. (2016)
Chromadorida				
* Euchromadora* sp.	PRJNA506150	Transcriptome	C: 39.7% [S: 24.6%, D: 15.1%], F: 2.9%, M: 57.4%	Smythe et al. (2019)
* Halichoanolaimus dolichurus*	PRJNSA787273	Transcriptome	C: 62.3% [S: 33.9%, D: 28.4%], F: 1.9%, M: 35.8%	Ahmed et al. (2022)
Desmodorida				
* Chromadoropsis vivipara*	PRJNA772260	Transcriptome	C: 68.6% [S: 21.4%, D: 47.2%], F: 2.9%, M: 28.5%	Tchesunov et al. (2023)
* Laxus oneistus*	PRJNA609171	Transcriptome	C: 70.2% [S: 54.6%, D: 15.6%], F: 3.4%, M: 26.4%	Paredes et al. (2022)
Araeolaimida				
* Dorylaimopsis* sp.	PRJNA767231	Transcriptome	C: 64.9% [S: 28.2%, D: 36.7%], F: 2.4%, M: 32.7%	Ahmed et al. (2022)
* Sabatieria punctata*	PRJNA953805	Genome	C: 58.5% [S: 39.5%, D: 19.0%], F: 5.0%, M: 36.5%	Lee et al. (2023)
Monhysterida				
* Linhomoeus* sp.	PRJNA953805	Genome	C: 37.1% [S: 28.6%, D: 8.5%], F: 3.8%, M: 59.1%	Lee et al. (2023)
* Paralinchomoeus* sp.	PRJNA953805	Genome	C: 40.2% [S: 33.0%, D: 7.2%], F: 4.0%, M: 55.8%	Lee et al. (2023)
* Theristus* sp.	PRJNA953805	Genome	C: 42.8% [S: 37.4%, D: 5.4%], F: 4.6%, M: 52.6%	Lee et al. (2023)
* Sphaerolaimus* sp.	PRJNA768956	Transcriptome	C: 51.3% [S: 15.6%, D: 35.7%], F: 2.5%, M: 46.2%	Ahmed et al. (2022)
* Gammarinema scyllae*	PRJNA767233	Transcriptome	C: 52.3% [S: 33.5%, D: 18.8%], F: 1.0%, M: 46.7%	Ahmed et al. (2022)
Plectida				
* E. matsi*	PRJEB79390	Genome	C: 60.6% [S: 60.1%, D: 0.5%], F: 4.7%, M: 34.7%	This work (neEchMats5.1)
* E. matsi*	PRJEB79327	Genome	C: 60.9% [S: 60.4%, D: 0.5%], F: 4.5%, M: 34.6%	This work (neEchMats11.1)
* E. matsi*	PRJEB97261	Genome	C: 54.5% [S: 54.1%, D: 0.4%], F: 4.0%, M: 41.5%	This work (neEchMatsONT)
* S. elegans*	PRJNA768632	Transcriptome	C: 31.0% [S: 18.3%, D: 12.7%], F: 4.6%, M: 64.4%	Ahmed et al. (2022)
* N. parasiticus*	PRJNA707491	Transcriptome	C: 58.5% [S: 33.9%, D: 24.6%], F: 2.8%, M: 38.7%	Ahmed et al. (2022)
* Anaplectus granulosus*	PRJNA506144	Transcriptome	C: 38.5% [S: 22.6%, D: 15.9%], F: 6.8%, M: 54.7%	Smythe et al. (2019)
* Plectus sambesii*	PRJNA390260	Genome	C: 75.9% [S: 49.2%, D: 26.7%], F: 5.0%, M: 19.1%	Rošić et al. (2018)
Rhabditida				
* Dracunculus medinensis*	PRJEB500	Genome	C: 78.3% [S: 77.7%, D: 0.6%], F: 5.8%, M: 15.9%	International Helminth Genomes Consortium (2019)
* Anguillicola crassus*	PRJNA81117	Transcriptome	C: 77.0% [S: 72.3%, D: 4.7%], F: 0.6%, M: 22.4%	Heitlinger et al. (2014)
* Gnathostoma spinigerum*	PRJNA502990	Transcriptome	C: 84.0% [S: 59.5%, D: 24.5%], F: 3.0%, M: 13.0%	Nuamtanong et al. (2019)
* Syphacia muris*	PRJEB524	Genome	C: 82.5% [S: 81.0%, D: 1.5%], F: 3.4%, M: 14.1%	International Helminth Genomes Consortium (2019)
* Acrobeloides nanus*	PRJEB26554	Genome	C: 78.8% [S: 60.1%, D: 18.7%], F: 2.3%, M: 18.9%	Schiffer et al. (2018)
* Bunonema* sp.	PRJNA655932	Genome	C: 25.3% [S: 24.9%, D: 0.4%], F: 2.7%, M: 72.0%	Casasa et al. (2021)
* Poikilolaimus oxycercus*	PRJNA758215	Genome	C: 54.0% [S: 51.5%, D: 2.5%], F: 5.2%, M: 40.8%	Collins et al. (2023)
* Rhabditoides inermis*	PRJEB71643	Genome	C: 70.6% [S: 63.6%, D: 7.0%], F: 5.3%, M: 24.1%	Rödelsperger et al. (2024)
Nematomorpha				
* Gordionus montsenyensis*	PRJEB63266	Genome	C: 33.4% [S: 22.4%, D: 11.0%], F: 0.9%, M: 65.7%	Eleftheriadi et al. (2024)
Priapulida				
* Priapulus caudatus*	PRJNA20497	Genome	C: 46.5% [S: 37.0%, D: 9.5%], F: 2.1%, M: 51.4%	Webster et al. (2006)

### Creation of figures

The 3 *E. matsi* genomes were mapped to the predicted rhabditid nematode ancestral linkage groups (Nigon elements) (Fig. 4) using https://pgonzale60.shinyapps.io/vis_alg/ (Gonzalez de la Rosa et al. 2021). BlobPlots were generated in *BlobToolKit* viewer v4.3.13 (Challis et al. 2020). Phylogenetic trees were visualized in *TreeViewer 2.2.0* (Bianchini and Sánchez-Baracaldo 2024). All bitmap images and photographs were edited using the *Affinity Photo 2* (https://affinity.serif.com/en-us/photo/), while all vector graphic illustrations and final images were created/edited using the *Affinity Designer 2* (https://affinity.serif.com/en-us/designer/).

## Results

### New data on the morphology of *E. matsi*

**Fig. 2.**
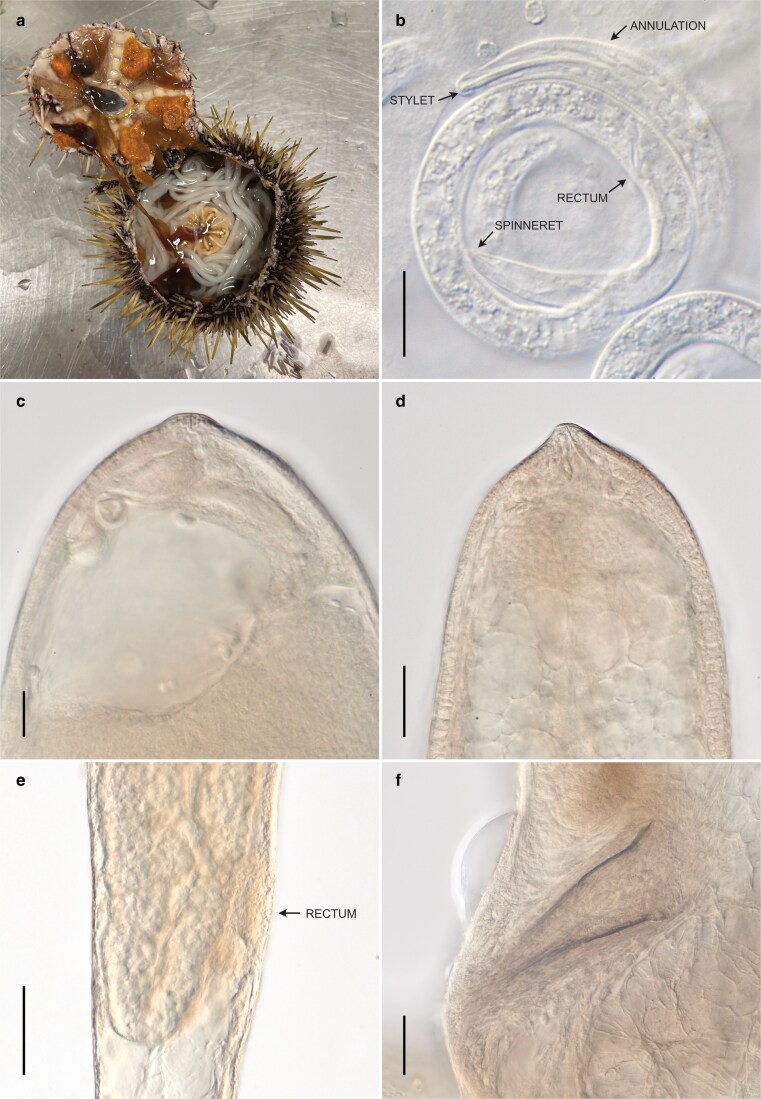
*E. matsi* Jones & Hagen, 1987. a) Nematodes inside the host. b) Juvenile showing a stylet, annulated cuticle, rectum, and spinneret. c and d) Anterior end of immature female individuals. e) Posterior end of a juvenile female showing atavistic rectum. f) Spicules of an adult male.

Adult and eggs of *E. matsi* were isolated from the body cavity of *Strongylocentrotus* sp. sea urchins in northern Norway (Fig. 2a). They were identified following the original description of the species (Jones and Hagen 1987), to which we can add some supplementary observations. Unhatched first-stage juveniles possess a distinctly annulated cuticle (Fig. 2b), which is also visible in Fig. 7D in Jones and Hagen (1987). We confirm that sensory structures of the anterior end are indistinct, mostly obscured by the eggshell. The stoma of the unhatched first-stage juveniles has the shape of a well-defined stylet gradually extending into the lining of the pharynx but is reduced in mature individuals (Fig. 2c and d). The rectum in the unhatched first-stage juveniles is well developed, opening to the exterior, and not vestigial (Fig. 2b). The spinneret and caudal glands in the unhatched first-stage juveniles are present and appear to be fully functional (Fig. 2b). Several immature females showed the remains of a rectum (Fig. 2e) appearing as a group of cells on the ventral body side, adjacent to the cuticle but without distinct lumen.

### The genome of *E. matsi*

**Fig. 3.**
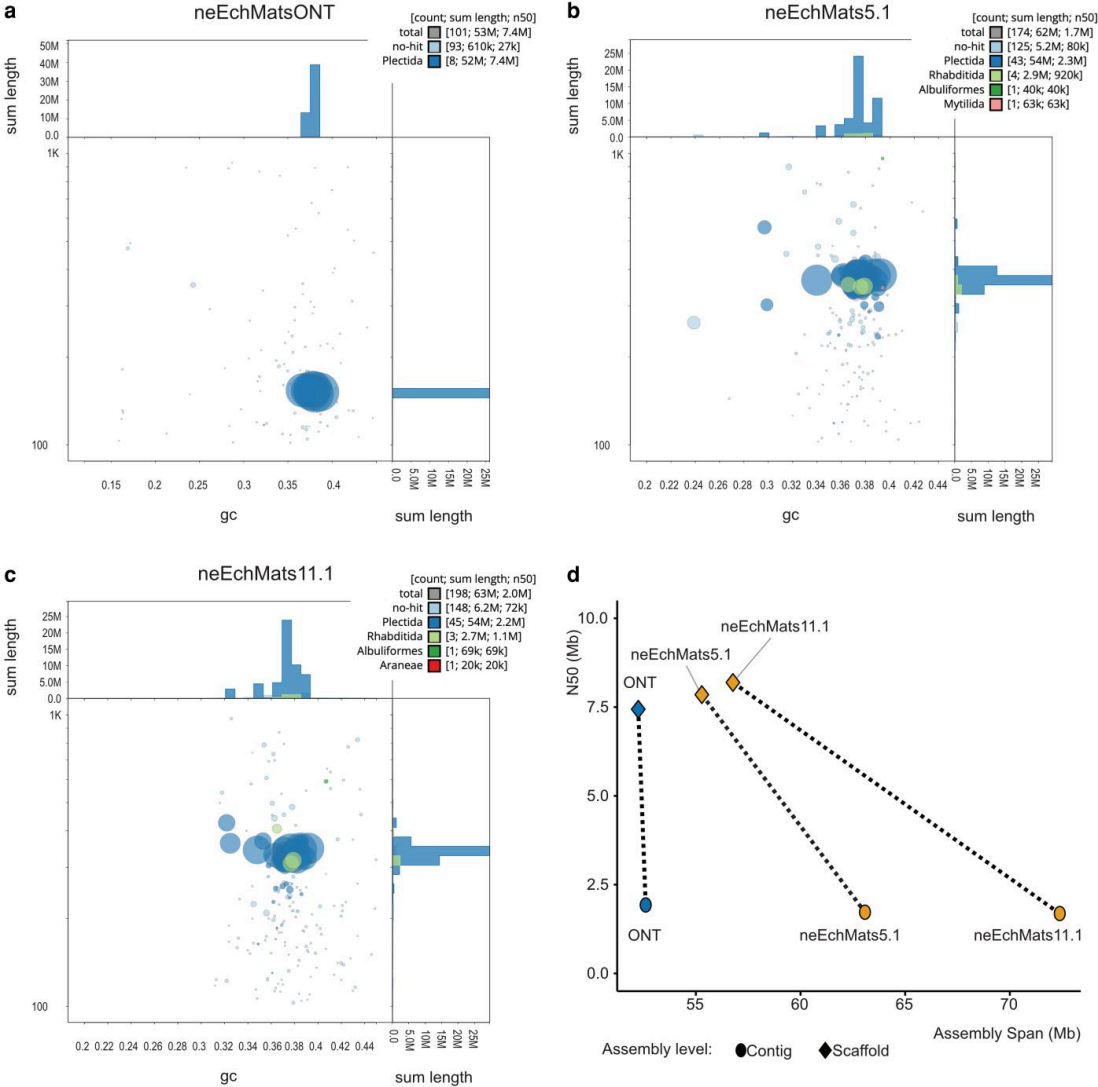
Genome assembly of *E. matsi*: a to c) BlobToolKit GC-coverage plots of primary assemblies. (a, neEchMats5.1; b, neEchMats11.1; c, neEchMatsONT). In each BlobToolKit plot, the assemblies are split into contigs, breaking scaffolds at stretches of 10 or more N residues. The *x* axis shows contig GC proportion (between 0.2 and 0.45), while the *y* axis shows coverage in the respective dataset. Note that coverage for neEchMatsONT is derived from mapping of the neEchMats5.1 PacBio HiFi reads. Contigs are represented by circles, with the diameter sized according to their span. Contigs are colored by the ordinal-level taxon of the best-matching BUSCO locus on each. While most contigs are designated “Plectida,” some have aberrant best-scoring mappings to other taxa. However, these mappings are not supported by more fine-scale analyses. d) Improvement in N50 and reductions in span on scaffolding with Hi-C data and removal of haplotypic duplication for the ONT (blue) and PacBio (yellow) assemblies.

**Fig. 4.**
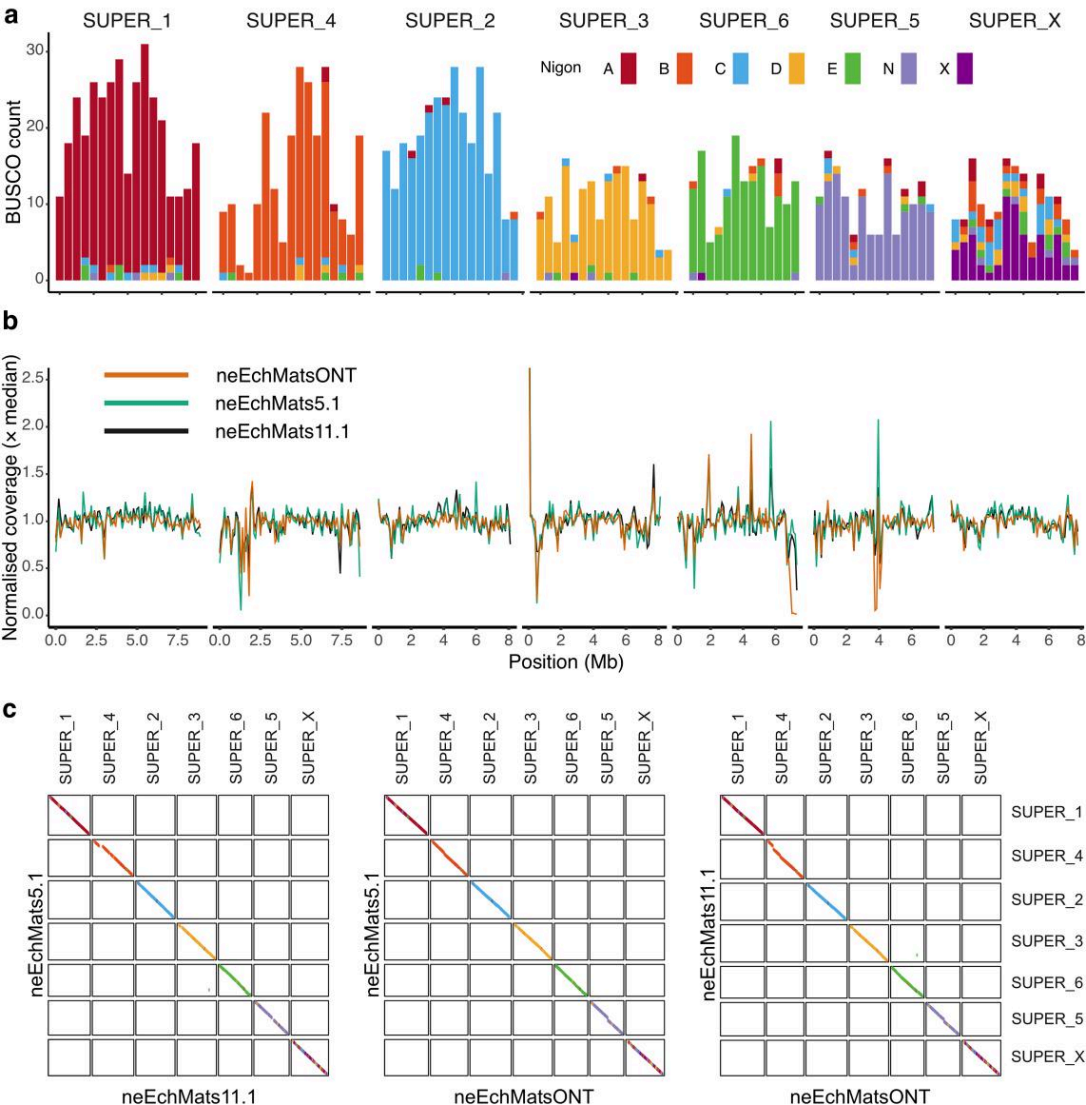
*E. matsi* retains the ancestral linkage groups of Rhabditida. a) The chromosomal pseudomolecules of neEchMats11.1 correspond to intact Nigon elements; for each chromosome, the count of nematoda_odb10 genes allocated to each Nigon element is plotted as a stacked histogram in 500-kb bins. b) Normalized read coverage for the data from the neEchMats5, neEchMats11.1, and neEchMatsONT samples. A *y* axis normalized coverage limit of 2.5 was set to reduce the impact of a collapsed tandem ribosomal array at the start of SUPER_3, which had almost 5-fold normalized coverage. c) Pairwise synteny comparisons of neEchMats5.1, neEchMats11.1, and neEchMatsONT. Each chromosomal pseudomolecule is colored by the Nigon element allocation of nematoda_odb10 BUSCO loci.

The 3 assemblies generated have spans ranging from 52.6 to 64.6 Mb, and each was scaffolded into 7 chromosomes (Fig. 3). The primary assembly of neEchMatsONT (Fig. 3a) was more contiguous than that of neEchMats5.1 or neEchMats11.1 (Fig. 3b and c), likely because the ONT data, having a longer read N50, was able to bridge longer repeats than was the PacBio HiFi, but was reduced in span in comparison with the PacBio assemblies (Fig. 3d). This is likely because the increased error rate of ONT over that of PacBio HiFi reads leads to reduced resolution of repeat regions. The higher accuracy and longer read N50s of PacBio sequencing allows assembly and phasing of both haplotypes alongside improved resolution of multimapping reads over repetitive or heterozygous regions. Both PacBio assembly spans were significantly reduced upon purging of the alternate haplotypes and scaffolding to chromosomal contiguity, but retention of repeats and heterozygosity resulted in a higher final assembly span than that of the ONT assembly (Fig. 3d). We note that even in the longest PacBio assembly, neEchMats11.1, longer repeats, such as the ribosomal RNA cistron, are still collapsed (Fig. 4a), and thus the true genome size will be larger than currently estimated.

Scaffolding with Hi-C resulted in resolved chromosomes for all datasets, increasing contiguity across all platforms with scaffold N50 (likely equivalent to chromosomal N50) converging around 7.8 Mb (0.4-Mb SD). The arrangement of the rhabditid nematode ancestral linkage groups (Nigon elements) (Fig. 4a) closely corresponds to the theoretical archetypal arrangement for Rhabditida (Gonzalez de la Rosa et al. 2021). Interestingly, the chromosome corresponding to Nigon X included many loci previously allocated to other elements (about 50% of the loci mapped to this chromosome), while other chromosomes were more clearly assigned to 1 Nigon element, with <10% of loci from other Nigon element-defining groups. This likely reflects the phylogenetic position of *E. matsi*, as Nigon elements were defined based on genomes from Rhabditida only. We presume this overrepresentation of loci assigned to other Nigon elements is because they migrated to those autosomal elements in the lineage leading to the last common ancestor of Rhabditida. Chromosomes of all 3 individuals were highly syntenic (Fig. 4b). The increased assembly span of neEchMats11.1 over the other PacBio assembly neEchMats5.1 may reflect greater heterozygosity inherited from more divergent parents, or genuine differences in genome sizes among individuals. The higher span of neEchMats11.1 is reflected in gaps in synteny with neEchMats5.1 and neEchMatsONT on both chromosomes corresponding to Nigon element B and element N (SUPER_3 and 5, respectively), both of which are also larger in neEchMats5.1 than the ONT assembly. One potential transposition was apparent among individuals, where a Nigon E BUSCO appeared uniquely on Nigon D for neEchMats11.1 (Fig. 4b).

Nigon X corresponds to the X chromosome in Rhabditid nematodes, where individuals are (usually) XX (female) and XO (male). We assessed read coverage of the chromosomes in our *E. matsi* read sets to identify a putative sex chromosome. The 2 individual nematodes sequenced (neEchMats5 and neEchMats11) showed no difference in coverage of any chromosome, identifying neEchMats11 as an immature female. In the pooled nematodes (females) sequenced to generate the neEchMatsONT assembly, no drop in coverage of any chromosome was observed either. Moreover, normalized read coverage followed similar trajectories across different sequenced individuals/samples and sequencing platforms.

### Phylogenetic position of *E. matsi*

BUSCO completeness was assessed using both BUSCO 5.5.0 with the *nematoda_odb10* gene set and BUSCO 6.0.0 (Tegenfeldt et al. 2025) with the *nematoda_odb12* and *metazoa_odb12* index gene sets (Table 2). Using *nematoda_odb10* with 3,131 reference genes derived from 7 genomes, the completeness of all 3 assemblies ranged between 61.0% and 61.3%, with only 0.4% to 0.5% of the genes duplicated (Table 2). This low BUSCO completeness is not concerning, as the BUSCO *nematoda_odb10* locus list is biased toward Rhabditida species, and species from lineages not included in the set used to define the list often score poorly. Completeness of all 3 genomes assessed with the *nematoda_odb12* with only 596 genes but based on a much expanded set of 56 genomes (albeit still biased toward clades III, IV, and V) ranged between 95.8% and 96.1% (Table 2), while the same stats assessed with *metazoa_odb12* varied between 78.7% and 79.2%.

**Table 2.** Assembly statistics.

Assembly	neEchMatsONT	neEchMats5.1	neEchMats11.1
Accession	GCA_977018575	GCA_964256745.1	GCA_964248945.1
Primary decontaminated assembly length (Mb)	52.6	63.1	72.4
Contig N50 (Mb)	1.93	1.72	1.69
Contig N	226	237	651
Scaffold assembly length (Mb)	52.2	55.3	56.8
Scaffold N50 (Mb)	7.44	7.85	8.19
Scaffold N	148	7	8
BUSCO nematoda_odb10 (3,131 genes from 7 genomes)			
Completeness	61.3%	61.0%	61.3%
Duplication	0.4%	0.5%	0.5%
BUSCO nematoda_odb12 (598 genes from 56 genomes)			
Completeness	96.1%	96.0%	95.8%
Duplication	1.2%	1.0%	1.5%
BUSCO metazoa_odb12 (672 genes from 206 genomes)			
Completeness	79.2%	78.7%	79.0%
Duplication	0.6%	0.9%	0.6%

**Fig. 5.**
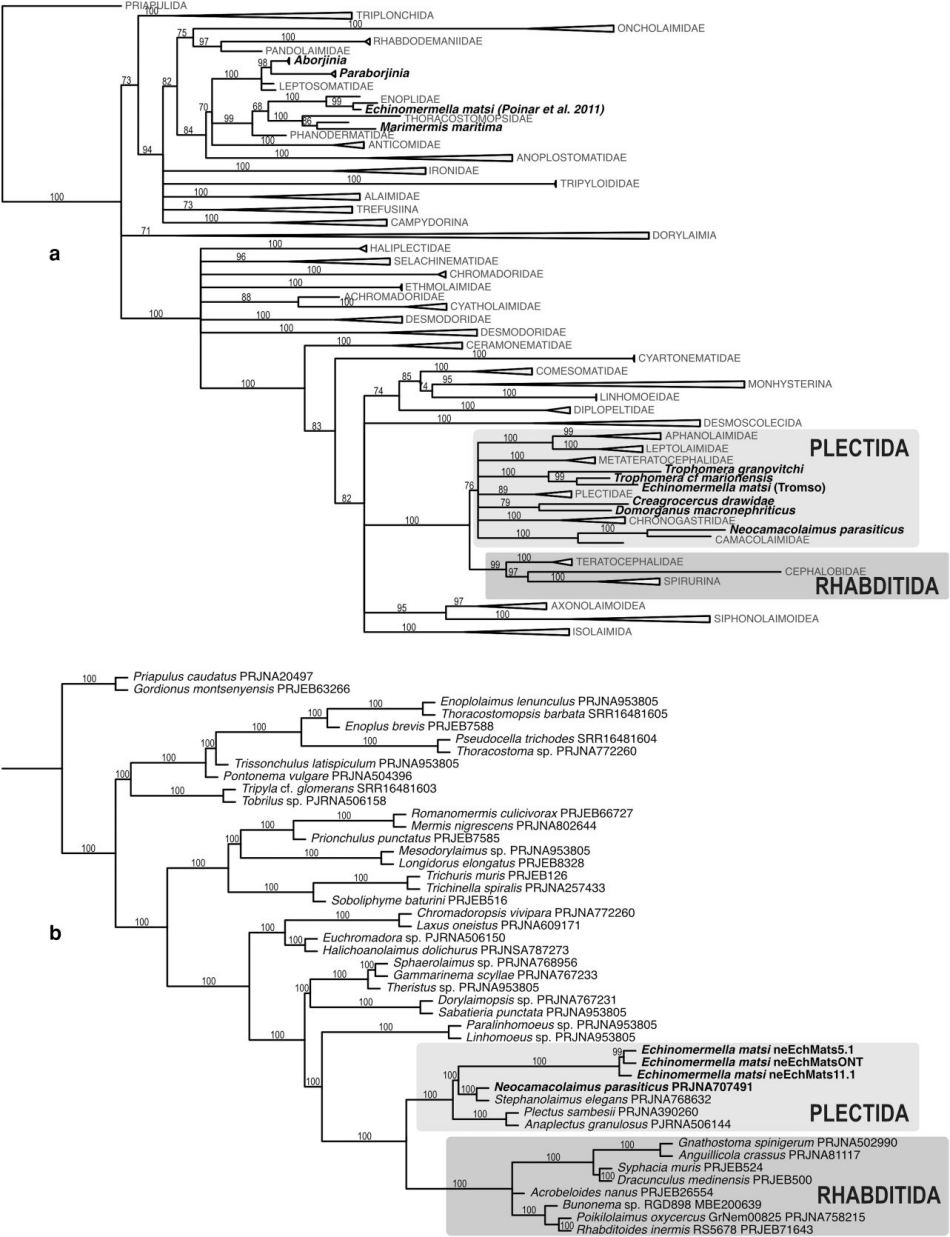
Phylogenetic relationships between *E. matsi* and other nematodes. Phylogenies were estimated using 18S rRNA (a) and multigene (b) datasets. Names in bold highlight nematode parasites of invertebrates (mostly marine) specifically discussed in the text.

We used full-length 18S rRNA and 1,307 *nematoda_odb10* BUSCO loci in phylogenetic analyses to place *E. matsi* in the wider Nematoda phylogeny. Both phylogenetic analyses firmly place *E. matsi* within Plectida (Fig. 5), contrary to the result obtained previously using a fragment of 18S rRNA putatively from *Echinomermella* (Poinar et al. 2011; Ahmed and Holovachov 2021; Westerman et al. 2021; Tchesunov et al. 2023). This fragment (Poinar's accession HQ668023) had no match in the *E. matsi* genome sequences and is likely to be a chance contaminant.

The differences in taxonomic composition between the 18S rRNA locus and protein-coding gene datasets result in potentially conflicting placements for *E. matsi*. The phylogenetic hypothesis based on the 18S rRNA locus includes more species and places *Echinomermella* as an ingroup of paraphyletic *Trophomera* (Fig. 5a), a parasitoid of marine invertebrates (Tchesunov and Rozenberg 2011), but the tree is not well resolved. The phylogeny based on 1,307 protein-coding gene trees (Fig. 5b) includes fewer species and is well resolved. *E. matsi* is placed as a sister to a clade of *Neocamacolaimus parasiticus*, another parasitoid of marine invertebrates (Holovachov and Boström 2014), and *Stephanolaimus elegans*, a free-living marine nematode (Ditlevsen 1919). *Neocamacolaimus* is placed in a separate clade from *Trophomera* in analyses based on 18S rRNA (Ahmed and Holovachov 2020). No genomic data are yet available for *Trophomera*, but formally the 2 analyses (18S rRNA and multi-gene) are not in conflict.

### Changes to the classification of nematodes

Poinar (2011) proposed a new family, Echinomermellidae, and a new order, Echinomermellida, within Enoplia (clade II) to accommodate this unusual parasite, in contradiction to the results of his phylogenetic analysis. Molecular phylogeny places the HQ668023 sequence (generated in Poinar et al. 2011) within the now paraphyletic genus *Enoplus* (Figure 3 in Poinar et al. 2011), which means that from strictly phylogenetic point of view, *Echinomermella* should be placed in the family Enoplidae, of the order Enoplida, and not in a separate family, Echinomermellidae, and the order Echinomermellida. Our 18S rRNA and multigene phylogenetic analyses place *Echinomermella* within the order Plectida with high support. Our 18S rRNA-based phylogeny places *Echinomermella* as a lineage within a paraphyletic *Trophomera* Rubtzov and Platonova 1974. Based on these results, we propose/confirm the following changes in the classification of the phylum Nematoda: (i) the genus *Echinomermella* Chitwood 1933 to be placed within the family Benthimermithidae Petter 1980; (ii) the family Echinomermellidae Poinar 2011 to be considered a junior subjective synonym of the family Benthimermithidae Petter 1980; (iii) the order Benthimermithida Tchesunov 1995 to be considered a junior subjective synonym of the order Plectida Gadea 1973; and (iv) the order Echinomermellida Poinar 2011 to be considered a junior subjective synonym of the order Plectida Gadea 1973.

## Discussion

We sequenced the genome of the enigmatic parasitic nematode *E. matsi* both to better illuminate the biology of this poorly understood marine species and to resolve conflicting concepts of the placement of *Echinomermella* within the diversity of Nematoda. Using both single-specimen PacBio HiFi sequencing and bulk-extract Oxford Nanopore sequencing, we found that these platforms were both able to generate high-quality primary assemblies, but that Hi-C chromatin conformation capture data was critical in resolving the assemblies to full chromosomal level. Importantly, we find that the 7 chromosomes of *E. matsi* correspond to the 7 ancestral linkage groups, or Nigon elements, predicted from analysis of the rearranged genomes of rhabditid nematodes. This finding strongly supports the model proposed by Gonzalez de La Rosa et al. (2021) that the last common ancestor of Rhabditida had 7 chromosomes and suggests that this ancestral linkage group model can be extended back to the last common ancestor of Plectida and Rhabditida.

**Fig. 6.**
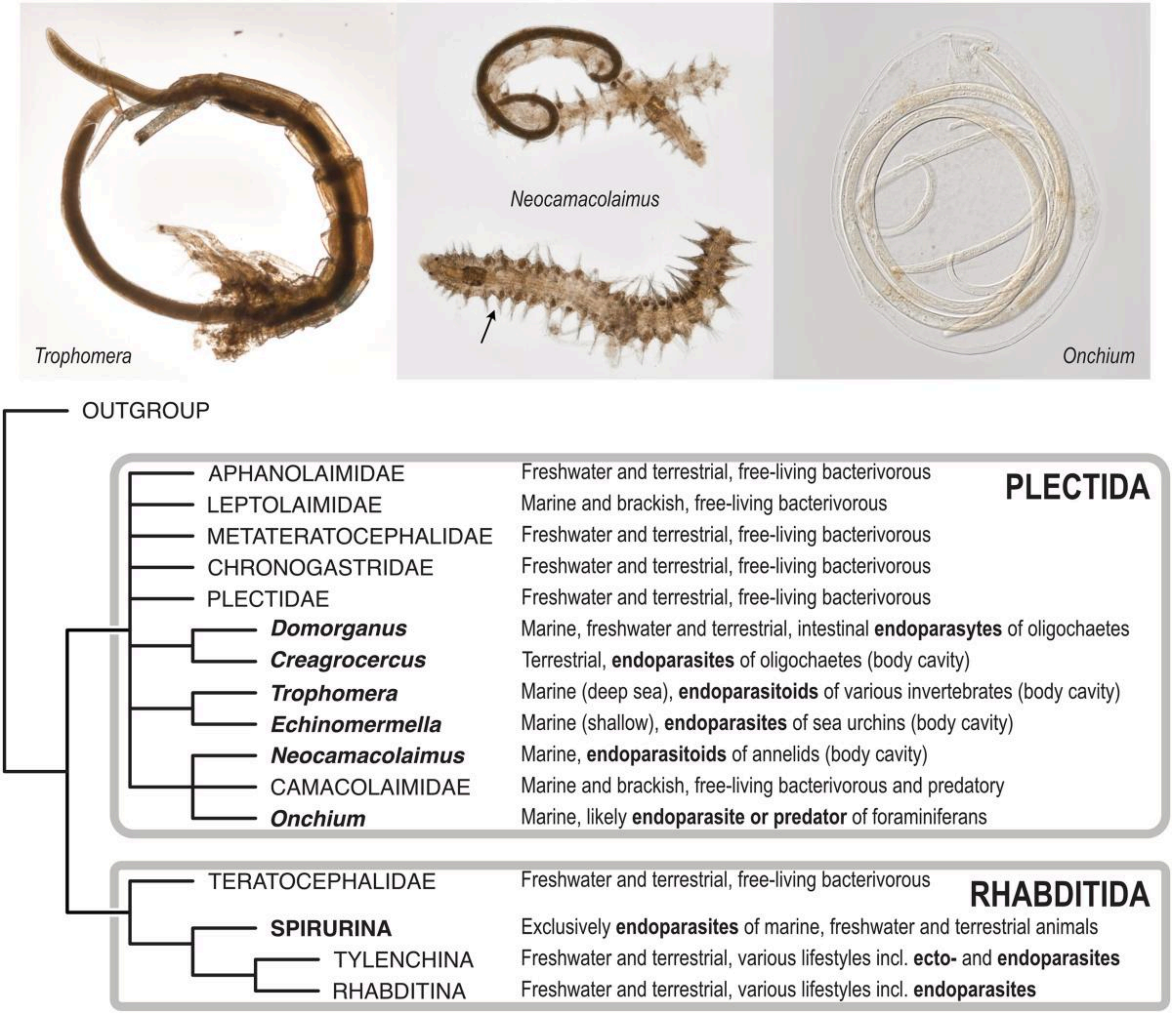
Simplified phylogeny showing known animal-parasitic lineages within Plectida. Parasites in the order Plectida and their relationships to free-living lineages and to Spirurina (clade III) are shown, with photographic depictions of *Trophomera*, *Neocamacolaimus*, and *Onchium* inside their respective hosts. Photographs by Oleksandr Holovachov.

*E. matsi* is robustly placed within the generally free-living order Plectida. Plectida includes both marine and fresh-water taxa, with the bulk of species being marine detritivores. With the addition of *Echinomermella*, Plectida now includes several distinct, independently evolved lineages that have parasitic relationships with invertebrates and protists. The following 3 monophyletic lineages can be characterized (Fig. 6):

1. A partially supported lineage uniting *Echinomermella* and *Trophomera.* Two additional monotypic genera, *Adenodelphis* Petter, 1983 and *Bathynema* Miljutin & Miljutina, 2009, are closely related to *Trophomera*. Both species of *Echinomermella* inhabit the body cavity of sea urchins as adults, and both have very limited distribution compared to the areas inhabited by their hosts. The other 3 genera can be classified as parasitoids, as they inhabit the body cavity of their hosts as juveniles but have nonfeeding, free-living adults. The precise identity of the host species is known only for some *Trophomera* species and for *Adenodelphis*; these host species include nematodes, polychaetes, priapulids, mollusks, holothuroids, and various crustaceans (Tchesunov and Ivanenko 2022).
2. A monophyletic clade uniting the genera *Creagrocercus* Baylis, 1943 (family Creagrocercidae) and *Domorganus* (family Ohridiidae). All 3 known species of *Creagrocercus* are endoparasites of terrestrial earthworms, inhabiting the coelomic cavity of the host, but nothing else is known about their ecology or interactions with the hosts (Ivanova and Spiridonov 2011). The biology of the genus *Domorganus* Goodey, 1946, with 10 described species, is somewhat better known. Although most of its species were found in marine and freshwater sediments and soil (Holovachov 2012), 3 are associated with several species of oligochaetes, inhabiting the intestine and possibly feeding on the gut microbiome (von Thun 1967; Valovaya 1989; Tchesunov and Sturhan 2004).
3. The family Camacolaimidae is represented mostly by free-living species, the feeding biology of which remains enigmatic, but there are 2 exceptions. *N. parasiticus* (Holovachov and Boström 2014) and a similar unnamed species (Tchesunov 2009) are parasitoids of polychaetes, where the juveniles develop in the coelomic cavity of the host, while adults (known only for *N. parasiticus*, and even then only males were found) are free living. Another, unrelated to *Neocamacolaimus*, group of species inhabit cells of foraminiferans, both testate and atestate, and include *Smithsoninema inaequale* (Hope and Tchesunov 1999), several species of *Onchium* (Holovachov 2015) and several unidentified and undescribed species of Camacolaimidae (Miljutin and Miljutina 2016; Fig. 6). Observations of fixed specimens suggest that these nematodes can reproduce and develop within the form cell, but nothing else is known about their biology.

Parasitism has originated many times in Nematoda (Blaxter and Koutsovoulos 2015), especially within Rhabditida. Plectida are the closest sister lineage to Rhabditida as a whole and just 1 node away from Spirurina (clade III). All species in Spirurina are parasites of animals, including important parasites of humans (the causative agents of filariasis and onchocerciasis) and farm animals (*Ascaris* large roundworms). While the phylogeny of Spirurina is not yet well resolved (Nadler et al. 2007), the earliest branching families are Gnathostomatidae and Anguillicolidae, Cucullanidae, Kathlaniidae, Quimperiidae, and Seuratidae (Ahmed and Holovachov 2021). All of these taxa use vertebrates as definitive hosts, while their intermediate hosts, when known, are crustaceans and fish. There are no known “intermediate” free-living forms with preadaptations to parasitism (Sudhaus 2010). While 2 groups, Oxyurida and Rhigonematida, are parasites of terrestrial insects, they are not the earliest-branching clades in Spirurina, although their morphology and biology in general retain many features of an “average” ancestral, terrestrial rhabditid (Sudhaus 2010).

One fundamental unanswered question is whether parasitism in Spirurina originally evolved in the marine environment or on land. Hypotheses explaining the origin of animal parasitism in terrestrial environments include preadaptations in bacterial-feeding, saprobiontic nematodes (Anderson 1984; Sudhaus 2010) that allow them to invade the intestine of a host, continue feeding on the gut microbiome where they withstand an anaerobic environment with higher osmotic pressure, and protect themselves from digestive enzymes (in addition to other factors discussed in detail by Sudhaus 2010). Marine invertebrates, as potential hosts, have 2 major differences from terrestrial counterparts (Tchesunov and Ivanenko 2022). Most marine invertebrates do not depend on the gut microbiome to break down their food, with the possible exception of littoral oligochaetes inhabited by *Domorganus* (von Thun 1967; Valovaya 1989). Secondly, the body cavity of marine invertebrates is almost isotonic compared to sea water. As such, the path toward animal parasitism in marine environments must involve very different physiological and genomic adaptations compared to the same process in terrestrial habitats.

Nematodes of the order Plectida include several genera and species with well-characterized genomes and transcriptomes (Rošić et al. 2018; Ahmed et al. 2022). Some terrestrial, nonparasitic Plectids can be maintained in the laboratory and are becoming satellite models for comparative genomics (Guiglielmoni and Schiffer 2024). Deeper study of Plectida, especially of the independent origins of parasitism within the order, thus represents an unparalleled opportunity to shed light on the origin and early evolution of Spirurina, the most diverse and economically important group of animal parasitic nematodes.

## Data Availability

Several complete and partial individuals of *E. matsi* are deposited both in ethanol and on permanent microscopic slides in the invertebrate collection of the Department of Zoology of the Swedish Museum of Natural History (accession numbers SMNH 224932–224953). Raw data used for PacBio assembly and scaffolding are available from the Sequence Read Archive (accession numbers for Revio ERX12753396, ERX12816597 and for Illumina NovaSeq X paired end ERX12760447–ERX12760450). Raw data used for ONT assembly are available from the European Nucleotide Archive (accession number ERX16283629). Assembled genomes are available from INSDC databases under BioProject PRJEB79327, accession numbers GCA_964256745.1 (neEchMats5.1) and GCA_964248945.1 (neEchMats11.1), and BioProject PRJEB97261, accession number GCA_977018575 (neEchMatsONT). Input, intermediate, and output files generated during phylogenetic analysis using single locus (18S rRNA gene) and during phylogenomic analysis using multiple protein-coding genes as well as a custom bash script written to run the latter analysis are deposited as a FigShare dataset at https://doi.org/10.6084/m9.figshare.31852987.
